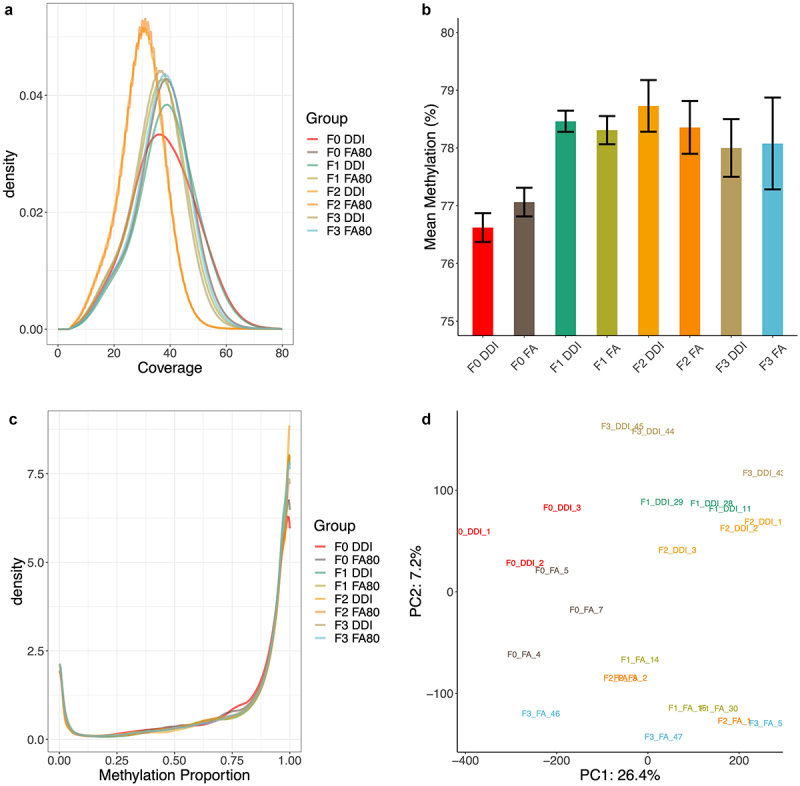# Correction

**DOI:** 10.1080/15592294.2024.2388387

**Published:** 2024-08-04

**Article title**: Folate-mediated transgenerational inheritance of sperm DNA methylation patterns correlate with spinal axon regeneration

**Authors**: Andy Madrid, Joyce Koueik, Ligia A. Papale, Roy Chebel, Isabelle Renteria, Emily Cannon, Kirk J. Hogan, Reid S. Alisch, and Bermans J. Iskandar

**Journal**: *Epigenetics*

**Bibliometrics**: Volume 19, Number 01, pages 1-17

**DOI**: https://doi.org/10.1080/15592294.2024.2380930

It has been noted by the authors that Figure 2 was incorrect in published article. The corrected images of Figure 2A, 2B, 2C, 2D has been placed below. This correction has not changed the description, interpretation, or the original conclusions of the article. The authors apologize for any inconvenience caused.

**Figure 2A-2D:**